# Behavioral responses around conspecific corpses in adult eastern gorillas (*Gorilla beringei spp.*)

**DOI:** 10.7717/peerj.6655

**Published:** 2019-04-02

**Authors:** Amy Porter, Winnie Eckardt, Veronica Vecellio, Katerina Guschanski, Peter Philip Niehoff, Urbain Ngobobo-As-Ibungu, Radar Nishuli Pekeyake, Tara Stoinski, Damien Caillaud

**Affiliations:** 1 The Dian Fossey Gorilla Fund International, Atlanta, GA, United States of America; 2 Animal Ecology, Department of Ecology and Genetics, Evolutionary Biology Centre, Uppsala Universitet, Uppsala, Sweden; 3 Institut Congolais pour la Conservation de la Nature, Kinshasa, Democratic Republic of Congo; 4 Department of Anthropology, University of California, Davis, Davis, CA, United States of America

**Keywords:** Grauer’s gorilla, Mountain gorilla, Thanatology, Kahuzi-Biega National Park, Volcanoes National Park, Behavior, Responses to death, Death

## Abstract

Humans were once considered unique in having a concept of death but a growing number of observations of animal responses to dying and dead conspecifics suggests otherwise. Complex arrays of behaviors have been described ranging from corpse removal and burial among social insects to quiet attendance and caregiving among elephants and primates. Less frequently described, however, are behavioral responses of individuals from different age/sex classes or social position toward the death of conspecifics. We describe behavioral responses of mountain gorillas (*Gorilla beringei beringei*) to the deaths of a dominant silverback and a dominant adult female from the same social group in Volcanoes National Park in Rwanda and the responses of Grauer’s gorillas (*Gorilla b. graueri*) to the corpse of an extra-group silverback in Kahuzi-Biega National Park, Democratic Republic of Congo. In gorillas, interactions between groups or with a lone silverback often result in avoidance or aggression. We predicted that: (i) more individuals should interact with the corpses of same-group members than with the corpse of the extra-group silverback; (ii) adult females with infants should avoid the corpse of the extra-group silverback; and (iii) in the mountain gorilla cases, individuals that shared close social relationships with the dead individual should spend more time with the corpse than other individuals in the group. We used a combination of detailed qualitative reports, photos, and videos to describe all occurrences of affiliative/investigative and agonistic behaviors observed at the corpses. We observed similar responses toward the corpses of group and extra-group individuals. Animals in all three cases showed a variety of affiliative/investigative and agonistic behaviors directed to the corpses. Animals of all age/sex classes interacted with the corpses in affiliative/investigative ways but there was a notable absence of all adult females at the corpse of the extra-group silverback. In all three cases, we observed only silverbacks and blackbacks being agonistic around and/or toward the corpses. In the mountain gorilla cases, the individuals who spent the most time with the corpses were animals who shared close social relationships with the deceased. We emphasize the similarity in the behavioral responses around the corpses of group and extra-group individuals, and suggest that the behavioral responses were influenced in part by close social relationships between the deceased and certain group members and by a general curiosity about death. We further discuss the implications close interactions with corpses have for disease transmission within and between gorilla social groups.

## Introduction

Cross culturally, humans engage in diverse post death practices, ranging from quiet observance to elaborate ceremonies (Irish, Lundquist & Nelsen, 1993; Lobar, Youngblut & Brooten, 2006). It was once widely assumed that humans were unique in caring for the dead and having a concept of death (Counts & Counts, 1991; Anderson, 2016). The growing field of comparative thanatology- the scientific study of death and dying among humans and other animals- has challenged this view by revealing complex responses to death among diverse organisms (social insects: Lopez-Riguelme & Fanjul-Moles, 2013; birds: (Iglesias, Stetkevitch & Patricelli, 2014; Swift & Marzluff, 2015); mammals: (Douglas-Hamilton et al., 2006; Anderson, 2011; Bercovitch, 2012; Reggente et al., 2016). Observational and experimental studies have described gatherings of animals around conspecific corpses accompanied by a variety of behaviors. Western scrub jays (*Aphelocoma californica*) have been described as holding “funerals”: upon detection of a dead conspecific or a similar sized heterospecific they start vocalizing, thereby attracting neighboring conspecifics who join the vocalization, turning the scene into a “cacophonous aggregation” (Iglesias, McElreath & Patricelli, 2012; Iglesias, Stetkevitch & Patricelli, 2014). Recruitment of conspecifics to the location of a corpse is believed to be a strategy whereby individuals acquire information about a salient danger in the environment. For example, the reaction of western scrub jays and American crows (*Corvus brachyrhynchos*) around conspecific corpses is similar to their reaction to a perceived predation risk, and they will avoid foraging at the site where the corpse was seen for up to 72 h (Iglesias, McElreath & Patricelli, 2012; Swift & Marzluff, 2015). American crows have also shown increased hippocampal and cerebellar activity while observing a person holding a dead conspecific (Cross et al., 2013).

Dead conspecifics may also be an indicator of the presence of pathogens. Many social insects have evolved elaborate behavioral strategies for corpse management and disposal that reduce exposure to contagion and epidemics (Lopez-Riguelme & Fanjul-Moles, 2013). Necrophobic behavior- the avoidance of dying and dead individuals- has been reported in insects (Yao et al., 2009; Lopez-Riguelme & Fanjul-Moles, 2013), sharks (*Carcharhinus* spp.; (Stroud et al., 2014), and small mammals (Prounis & Shields, 2013), and is believed to be an adaptive strategy to reduce exposure to disease (Wilson-Rich et al., 2009).

In contrast to necrophobic behavior, post-mortem inspection, such as sniffing, licking, touching, and grooming have been described in social insects (Lopez-Riguelme & Fanjul-Moles, 2013), giraffes (*Giraffa* spp.; (Bercovitch, 2012), cetaceans (Dudzinski et al., 2003; Reggente et al., 2016), primates (Boesch & Boesch-Achermann, 2000; Walsh et al., 2007; Anderson, 2011; Buhl et al., 2012; Stewart, Piel & O’Malley, 2012; Campbell et al., 2016; Yang, Anderson & Li, 2016), and elephants (*Loxodonta* spp.), the latter being especially well known for visiting and handling the bones of group and extra-group members (Douglas-Hamilton et al., 2006; McComb, Baker & Moss, 2006). Chimpanzees (*Pan troglodytes*) have even been observed using tools to groom the corpse of a group member (Van Leeuwen et al., 2016; Van Leeuwen, Cronin & Haun, 2017). Several explanations for these behaviors have been proposed, ranging from curiosity (Li et al., 2012) to compassion and grief (Douglas-Hamilton et al., 2006; Anderson, Gillies & Lock, 2010).

Among primates, most descriptions of responses to death give accounts of interactions of mothers and other group members with dead infants (Warren & Williamson, 2004; Biro et al., 2010; Cronin et al., 2011; Fashing et al., 2011). One of the most widespread and frequently described behaviors is mothers carrying and grooming their dead infant for prolonged periods, ranging from a few hours to >48 days (Warren & Williamson, 2004; Biro et al., 2010; Fashing et al., 2011; Cronin et al., 2011; Watson & Matsuzawa, 2018). Cold or dry climates can slow the process of decomposition and likely contribute to longer bouts of carrying (Fashing et al., 2011). Mothers have also been observed carrying corpses that have transformed into putrefying tissues, even at the cost of being stung by necrophagous insects (Perry & Mason, 2008). These observations are especially interesting given that exposure to putrescine, a pungent chemical compound emitted from decaying tissues, causes necrophobic behavior in humans (Wisman & Shrira, 2015) and other animals (Prounis & Shields, 2013). Infant corpses also elicit allomaternal-like behavior, such as carrying and grooming the corpse by group members other than the infant’s mother (Warren & Williamson, 2004; Fashing et al., 2011; Watson & Matsuzawa, 2018). There is some suggestion that mothers of dead infants are aware of the difference with live infants, as they carry the corpse or behave in atypical ways. For example, a white-faced capuchin (*Cebus capuchinus*) mother had her dead infant fully submerged in water while she drank (Perry & Mason, 2008). Corpses are also sometimes carried in the mouth or dragged by an arm or leg (Fashing et al., 2011; Cronin et al., 2011), which is believed to be a more efficient way to transport a corpse (Lopez-Riguelme & Fanjul-Moles, 2013).

Less frequently described are the behavioral responses of primates toward the death of conspecifics from different age/sex classes, social status, social relationships, circumstances of death, or from different social groups. A study on the behavioral responses of a group of chimpanzees to a dead adult female community member noted that females tended to be more fearful of the corpse whereas males showed more curiosity and interaction (Stewart, Piel & O’Malley, 2012). In another case, adult chimpanzees prevented infants from approaching the corpse (Boesch & Boesch-Achermann, 2000). Observations from Barbary macaques (*Macaca sylvanus*) indicated that the deaths of a high-ranking female and a juvenile male received nearly group-wide attendance while the deaths of the alpha male and an infant attracted only a few individuals (Campbell et al., 2016). However, it was unclear if the different behavioral responses were influenced by the deceased’s identity (age/sex class and social rank), the circumstances surrounding the deaths, or more likely, a combinations of factors. In a captive group of western lowland gorillas (*Gorilla gorilla gorilla*), the death of the silverback was followed by a significant increase in aggressive displays by adult females for three months after his death (Hoff, Hoff & Maple, 1998). Among chimpanzees, traumatic deaths (e.g., predation events, lethal accidents) have been described to elicit agitated responses of loud alarm calling and aggressive displays (Teleki, 1973; Boesch & Boesch-Achermann, 2000), while the demise of a sick and elderly female induced a more subdued response (Anderson, Gillies & Lock, 2010). In gelada baboons (*Theropithecus gelada*) a nulliparous adult female was observed carrying and repeatedly grooming a dead infant from a different social group (Fashing et al., 2011).

Understanding the nature and degree of variation in responses to conspecific death will help build a better perspective on how animals perceive and process death. Furthermore, understanding the extent to which individuals engage with conspecific corpses can have important implications for understanding disease transmission. Here we describe the behavioral responses of mountain gorillas (*Gorilla beringei beringei*) toward the deaths of a dominant group silverback and a dominant adult female and of Grauer’s gorillas (*Gorilla beringei graueri*) around the corpse of an extra-group silverback. Our descriptions are also supported with photographs and videos. In both the mountain gorilla cases, the deceased were dominant and established members of their social group whereas in the case with the Grauer’s gorilla, the extra-group silverback was an unrelated and presumably unknown individual to the group that encountered his corpse (see more in Group Histories below). In gorilla societies, most encounters between groups or between groups and lone silverbacks involve either avoidance or aggression with or without physical contact from silverbacks and sometimes blackbacks (Harcourt & Stewart, 2007). Adult females with young infants typically avoid extra-group silverbacks given the prevalence of infanticide (Harcourt & Stewart, 2007).

In light of the different social dynamics between group members and between extra-group competitors (e.g., an unrelated silverback) we predicted that (i) more individuals would engage with the corpses of intra-group members compared to the extra-group silverback and (ii) adult females with young infants would avoid the corpse of the extra-group silverback. Furthermore, in the two intra-group mountain gorilla cases, we predicted that (iii) individuals that shared close social relationships with the deceased would spend more time with the corpse than will other individuals in the group.

## Materials & Methods

### Study sites, group history, and background on dead individuals

#### Group titus: deaths of mountain gorilla silverback Titus and adult female Tuck

Field observations and data collection on mountain gorillas (*Gorilla beringei beringei*) were made by trackers and researchers from the Dian Fossey Gorilla Fund International’s Karisoke Research Center as part of a long-term research program (events reported here occurred in September 2009 and September 2010). All mountain gorilla observations where conducted in Volcanoes National Park, Rwanda (29.50°E, 1.49°S).

Titus, a dominant silverback mountain gorilla, was born on 24 August 1974 and monitored his entire life until his death on 14 September 2009, at the age of 35, in the eponymous group (Table 1). He became the dominant silverback of Group Beetsme in 1992. After the fission of Group Beetsme into Group Kuryama and Group Titus in 2007, Titus remained the dominant silverback in Group Titus until his death.

**Table 1:** Mountain and Grauer’s gorilla group compositions. The last three columns indicate the identity of the individuals of each age/sex class present on the days the three events were observed.

**Class/sex**	**Age (years)**	**Group titus individuals** r **(Titus’ death)**	**Group titus individuals(Tuck’s death)**	**Group chimanuka individuals**
Dominant silverback	>12	Titus	Umushikirano	Chimanuka
Subordinate silverback	>12	Umushikirano		
Adult female	>8	Tuck	Tuck	Siri
			Umwana	Mwinja
				AF3
Blackback male	8–12	Turakora	Turakora	BB1
		Pato	Pato	Pilipili
		Urwibutso	Urwibutso	Uhuru
				Nabanga Nshobole Busasa Numbi
Subadult female	6–8			JV1
Subadult male	6–8			Meteo JV2
Juvenile female	3.5–6			Koko
				Pori
Juvenile male	3.5–6	Segasira	Segasira	Karibu
		Ihumure		Marhale EF1
Infant female	0–3.5			Mulenge
Infant male	0–3.5			Mwira

Tuck, a dominant adult female mountain gorilla, was born in May 1972 and was monitored her entire life until her death on 6 September 2010, at the age of 38, in Group Titus. Tuck transferred from Group 5 to Group Beetsme in 1988 and remained with Titus after the fission of Group Beetsme.

#### Group Chimanuka: corpse of an extra-group Grauer’s gorilla silverback

Field observations and data collection on Grauer’s gorillas (*Gorilla beringei graueri*) were made by trackers and researchers of the Institut Congolais pour la Conservation de la Nature (ICCN) and the Dian Fossey Gorilla Fund International in June 2016 in the high-altitude sector of Kahuzi-Biega National Park, Democratic Republic of Congo (28.71°E, 2.30°S).

Group Chimanuka formed in 2002 when two adult females joined lone silverback Chimanuka. On 28 June 2016, the group (Table 1) discovered the corpse of an extra-group silverback unknown to the Park’s field staff or to on-site researchers. The cause of death was unknown and the corpse was estimated to be less than 24 h old because there were no insects on or near the body. No odor and no bloating of the body could be detected. The corpse was emaciated but there were no observable wounds. The skin was superficially peeled back in a 25 × 30 cm section on the right side of the mid back. The absence of signs of bleeding indicates that the lesion likely occurred post-mortem before Group Chimanuka discovered the corpse.

### Data collection

Focal-animal sampling data were collected in group Titus during the two 12-month periods prior to Titus’ and Tuck’s deaths (Altmann, 1974). Three or four focal individuals were selected at random among the visible individuals on each observation day (number of observation days: N_titus_ = 90 and N_tuck_ = 154) and were followed for 50min each. The total number of hours of observation was 252.5 h for the 12-month period prior to Titus’ death and 494.2 h for the 12-month period prior to Tuck’s death. The total number of hours of observation, per individual, ranged between 23.3 h and 154.2 h (mean = 36.1 h and 70.6 h, for the Titus and Tuck data collection periods, respectively). Affiliative social relationships in Group Titus were assessed using rates of grooming bouts per hour of focal-animal sampling, for each dyad before the deaths of Titus and Tuck. A grooming bout was defined as an individual picking through the hair of another individual with fingers or lips. If the groomer resumed grooming after any intervening activity, including resting, of 1 min or longer, then a separate grooming bout was recorded. In addition, dominance hierarchies in Group Titus were established using frequencies of displacements (recorded when one individual moved away from another individual as a direct response and within 5 s of the approach of another individual within 2 m) and avoidances (recorded when one individual moved away from another individual as a direct response and within 5 s of the approach of individual within 5 m) observed during focal-animal samples the 12 months before the deaths of Titus and Tuck.

At both field sites, observations of the group’s behavior in response to the corpses were made approximately 7–15 m from the corpses. Data were recorded using a combination of qualitative descriptions of all occurrences of affiliative, investigative, and agonistic behaviors (Table S1) of all animals within view (∼10–15 m) of the corpses, and photographs and videos, which are available on Figshare (see links below).

Individuals in both study groups were identified through a combination of nose prints, ear shape, scars, body size, and other physical features and were sexed both morphologically and genetically. The age class of Grauer’s gorillas was determined using morphological criteria (Harcourt et al., 1980; Watts, 1990). Identities were also verified with photographs and video. At Karisoke, group member relatedness was estimated from the long-term demographic data for maternal relatedness and in many cases paternity was also inferred from genetic analysis (Vigilant et al., 2015).

To investigate if the dead extra-group silverback was closely related to any of the members of Group Chimanuka, we genotyped fecal samples from all group members and the deceased individual at 12 variable microsatellite loci (vWF, D1s550, D4s1627, D5s1457, D5s1479, D6s474, D6s1056, D7s817, D8s1106, D10s1432, D14s306, and D16s2624) and the sex locus (Bradley, Chambers & Vigilant, 2001). We used PowerSoil Extraction Kits (QIAGEN) to extract DNA of fecal samples collected following the two-step method (Nsubuga et al., 2004). Genotyping followed the multiplex approach from Arandjelovic et al. (2009) with PCR conditions and electrophoresis setting as described in Baas et al. (2018). To obtain population-level allele frequencies, we produced genotypes from an additional 149 samples corresponding to 13 putative social gorilla groups from Kahuzi-Biega National Park. All these additional samples were collected from the night nests of unhabituated individuals. These samples correspond to roughly half of the estimated total gorilla population (Spira et al., 2016). We performed extensive PCR replications following established settings to guard against genotyping errors caused by allele dropout (Baas et al., 2018). Homozygous genotypes at a given locus were confirmed by a minimum of three and a maximum of 12 replicate PCRs. In addition, we confirmed that genotypes of known relatives (i.e., mother-offspring pairs inferred from behavioral observations in the habituated Group Chimanuka) were genetically compatible.

We used CERVUS v 3.0.7 (Kalinowski, Taper & Marshall, 2007) to calculate the observed number of alleles (A), observed (H_O_) and expected (H_E_) heterozygosity for each locus (Table S2), and to perform parentage analysis. None of the loci were in Hardy-Weinberg disequilibrium and therefore the full dataset was used for relatedness estimates. Pairwise relatedness was calculated for the dead extra-group silverback and all members of Group Chimanuka using the maximum-likelihood approach implemented in ML-RELATE (Kalinowski, Wagner & Taper, 2006) based on genotype and allele frequencies of 96 unique individuals from Kahuzi-Biega National Park. ML- RELATE takes into account the presence of null alleles, which improves the estimates compared to other approaches, and calculates Wright’s (1922) coefficient of relatedness (*r*), which ranges on the absolute scale between 0 and 1 and is expected to approach 0 for unrelated individuals and 0.5 for first-order relatives (e.g., parent–offspring or full siblings).

The study was conducted in compliance with legal requirements of the Republic of Rwanda and Democratic Republic of Congo. The data collection protocols were approved by the Rwandan Development Board and the Institut Congolais pour la Conservation de la Nature.

### Recovery/burial of corpses

In all three cases, the corpses were removed and/or buried after group members remained more than 100 m away for at least one hour. Removal and burial decisions were made in an effort to balance allowing sufficient time for the animals to engage with the corpses, preventing possible disease transmission, and performing post-mortem necropsies to maximize the chance of detecting the cause of death.

## Results

### Death of mountain gorilla dominant silverback, Titus

On 22 August 2009, three weeks prior to Titus’ death, 17-year-old lone silverback Umushikirano, confirmed through DNA analyses to be Titus’ son (Vigilant et al., 2015), joined the group. Umushikirano had been a solitary silverback since June 2007. He began making sexual overtures toward Tuck, the only adult female in the group at the time, despite her aggressive retaliations, which were often supported by other members in the group. After Umushikirano’s arrival, Titus and his group traveled long distances every day for three weeks in what appeared to be an attempt to elude Umushikirano, who persisted and gradually integrated into the group. During this time, the members of Group Titus, together with Umushikirano, interacted once with another group and twice with another lone silverback. Juvenile male, Ihumure, was severely wounded during these interactions (see ‘Discussion’). After three weeks, Titus started showing signs of weakness and the group stopped traveling.

On each of the five days before Titus’ death, Umushikirano was observed pursuing and displaying toward Tuck. Titus made cough grunt vocalizations towards Umushikirano but appeared too weak to perform more physically engaging displays/retaliations. Umushikirano was also aggressive toward Titus on two occasions, chest-beating and hitting him on the back. Titus was too weak to respond, but Tuck and her juvenile son, Segasira, aggressively chased off Umushikirano. Each day, Titus remained progressively closer to his nest (<30 m). Blackback Pato and the severely-injured Ihumure groomed Titus, and all group members remained within 50 m of him. Ihumure consistently remained close to Titus and shared his night nests. Their close association, including shared night nests, began six months prior, after Ihumure’s mother transferred groups. By the fifth day, Titus was able to move only a few meters from his nest and his condition became critical. The group moved only a few meters away from him while feeding.

**Figure 1:**
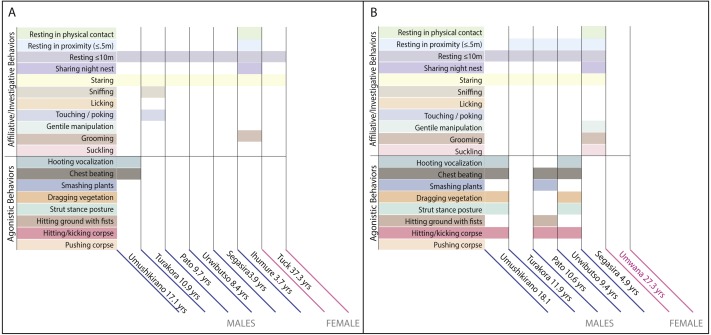
Distribution of social behaviors among individuals of different age/sex classes at same-group conspecific corpses. (A) Behaviors observed around the corpse of mountain gorilla silverback “Titus”. (B) Behaviors observed around the corpse of mountain gorilla adult female “Tuck”. Definitions of the behaviors are available in Table S1.

On 14 September 2009, field staff found Titus dead in his nest in prone decubitus with Ihumure resting in contact with his corpse. The other group members were not in sight. Trackers located the rest of the group 2.5 h later and found the animals separated. Umushikirano and the three blackback males of the group (Fig. 1, Table 1) were travelling together 100 m from Titus’ corpse, and Tuck and juvenile Segasira were travelling together 200 m from Umushikirano and the three blackbacks. Umushikirano made frequent hooting vocalizations followed by chest beats and Tuck appeared to be avoiding him by moving away from the sounds of his displays.

The group remained separated throughout the day but all of the individuals visited Titus’ corpse. Ihumure stayed by himself and was mostly in contact/proximity (<0.5 m) to Titus’ corpse, but he moved up to 50 m away to feed or when the other animals approached. Tuck and Segasira stayed together and within 150 m of Titus’ corpse. They approached Titus’ corpse once and sat a few meters away and stared at it. After nine minutes they moved off together as Umushikirano and the three blackbacks were heard approaching the area. Umushikirano and the three blackbacks stayed together within 50 m of Titus’ corpse.

Umushikirano and the three blackbacks visited Titus’ corpse twice. On the first visit, they all sat down and rested a few meters from the corpse. Blackback Turakora approached the corpse, touched and sniffed the back while the others continued to rest quietly 2 m away. After several minutes all of the animals left the corpse. Umushikirano continued to make frequent hooting vocalizations, occasionally followed by chest beats throughout the day. By 16:00, Umushikirano and the three blackbacks returned to Titus’ corpse a second time and rested 10 m from it. Ihumure stayed 30 m away from his corpse. The location of Tuck and Segasira was not known but they were likely close by. The trackers left Titus’ corpse in the forest overnight because the animals were still engaging with the corpse.

The following day at 08:16, field staff returned to Titus’ corpse and found Umushikirano, the three blackbacks, and Ihumure gathered around it. Umushikirano was staring at it from 4 m away while the others were resting quietly 10–15 m away. Although Tuck and Segasira could not be located, trackers found evidence that they nested 30 m from Titus’ corpse during the night. Umushikirano and the three blackbacks nested 15 m from Titus’ corpse and Ihumure slept in the nest with the corpse. After 45 min, the animals moved 100 m away to feed. Umushikirano made 23 hooting vocalizations while the others were feeding. At 13:00, all animals were >100 m away, and field staff recovered Titus’ corpse.

### Death of mountain gorilla dominant adult female, Tuck

On 16 August 2010, almost one year after the death of Titus, Tuck began showing signs of illness/weakness, including dramatically reduced feeding and lethargy. On 5 September 2010, she was often trembling, her legs were shaking, and she was unable to keep up with the group. Her blackback son, Urwibutso, and her juvenile son, Segasira, stayed close to her and rested in physical contact with her. Segasira moved back and forth between Tuck and the rest of the group.

On 6 September 2010 at 08:00, field staff discovered Tuck dead, lying on her back a few meters from the group’s nesting site. Segasira, Urwibutso, and the other two blackbacks were quietly gathered around her corpse staring at it. Umushikirano and the adult female Umwana, who joined Group Titus on 16 January 2010, were 100 m away. Segasira shared Tuck’s night nest and remained resting in contact with her corpse until the late morning. He was observed lying and sitting on her corpse, staring at her face, and he gently tried to move her head with his hands. He groomed her corpse and even attempted to suckle for a few seconds despite already being weaned (DOI: https://doi.org/10.6084/m9.figshare.6198584; DOI: https://doi.org/10.6084/m9.figshare.6198587).

Throughout the day, Tuck’s son Urwibutso displayed nine times; making hooting vocalizations and chest beating over Tuck’s corpse and one time he kicked her corpse in the abdomen and then stood in a strut stance posture (DOI: https://doi.org/10.6084/m9.figshare.6198590). One minute later, he dragged vegetation but then laid down <1 m from her corpse. Blackback Pato displayed twice; chest beating, smashing plants, hitting the ground, and hitting Tuck’s corpse in the abdomen with his fists. Umushikirano also displayed twice; during the first display, he dragged vegetation and hit Tuck’s corpse in the abdomen with his fists, and then stood in a strut stance posture. After his display, Pato and blackback Turakora embraced and mounted in a ventral-ventral position (DOI: https://doi.org/10.6084/m9.figshare.6198593). During the second display, he made a hooting vocalization and chest beat near her corpse. The vegetation around Tuck’s corpse was completely matted down as a result of intense displays. Between displays, however, the animals often rested quietly 0.5–10 m around her corpse while staring at it. The field staff left the corpse in the forest overnight, as most group members were still close by.

The following day at 08:00, field staff arrived at Tuck’s corpse and all of the group members were quietly resting within 10m from it. The group nested within 50m from her corpse. After 20 min, the group began to move away. At 10:00, all animals were feeding 200 m away, and field staff recovered Tuck’s corpse.

### Death of extra-group Grauer’s gorilla silverback

On 28 June 2016 at 11:10, field staff located Group Chimanuka feeding on the ground and in trees. The group was spread out and only eight of the 21 group members were in view. Twenty-four minutes later, the animals moved behind dense vegetation for a few minutes and when they were in view again, 17 of the 21 group members were observed gathered around the corpse of an extra-group silverback in prone decubitus with the head resting to the side. The three adult females and a two-year-old infant male were not present.

For 17 min, the animals were frequently shifting around the corpse, staring at it from different angles, sniffing and licking it, especially the section on the back where the skin was exposed. The silverback Chimanuka frequently stood in a strut stance posture 0.5–5 m from the corpse. On two occasions, he stood up on two legs, chest beat, charged directly over the corpse, hit the back with his fists, and then sat 2–5 m away from the corpse and stared at it (DOI: https://doi.org/10.6084/m9.figshare.6198596).

Many of the juveniles, sub-adults, and blackbacks groomed the corpse from different sides, concentrating mostly along the back and the posterior end. In addition to grooming, juveniles, sub-adults, blackbacks, and the silverback poked the corpse and then licked their fingers. They also licked the corpse (DOI: https://doi.org/10.6084/m9.figshare.6198599). There was a lot of movement around the corpse as animals frequently shifted positions, moving away then coming back, but they all generally stayed within 10m from the corpse (DOI: https://doi.org/10.6084/m9.figshare.6198602). When the animals were not grooming or inspecting the corpse they were generally resting quietly within 2–10 m of the corpse. One of the blackbacks, named BB1, groomed the corpse for extended periods, with one bout lasting five consecutive minutes on the back where the skin was exposed (DOI: https://doi.org/10.6084/m9.figshare.6198605).

Only one of the three adult females was observed near the corpse. She approached it with her two-year-old infant on her back and stared at it from <0.5m and within 0.5 m of Chimanuka but never touched or sniffed it. She left the site after less than one minute while carrying her infant on her back.

By 11:51, Chimanuka was inspecting the anal region of the corpse with his fingers and mouth and occasionally licked his fingers and lips. It was unclear if Chimanuka made some type of vocalization but all of the animals that were gathered around the corpse quickly parted, Chimanuka stood up on two legs and chest beat, and then pushed the corpse which rolled down the slope and landed ∼8m away on its back (DOI: https://doi.org/10.6084/m9.figshare.6198599).

The gathering of animals around the corpse was notably different after it rolled. Fewer individuals were present at the corpse and never more than five animals at a given time, compared to the majority of animals continually gathered at the corpse before it moved. Five blackbacks, including BB1, one sub-adult male, one sub-adult female, two juvenile males, and one juvenile female continued to stand over the corpse, alternately staring at it, sniffing, licking, grooming, and poking it then licking their fingers. Chimanuka remained within several meters of the corpse and displayed six times, making hooting vocalizations, chest beating, hitting the corpse with his fists, and displacing the animals near the corpse. His displays came in quick succession, with each display separated by 2–4 min.

BB1, another blackback, and a juvenile female returned to the site where the corpse had been before it rolled, sniffed the ground, and inspected the vegetation with their hands and mouths. There was a smashed pile of feces where the corpse had been lying.

After 38 min, all the animals moved >25 m from the corpse but BB1 continued to sit 10 m away. It was unclear when exactly BB1 left the vicinity, but he was the last animal to leave the corpse. By 14:00, all the animals were feeding and resting 100 m from the corpse. The corpse was left in the forest that night and buried at the site of death after a necropsy was performed on 30 June 2016.

Parent-offspring relationships between the extra-group silverback and any member of the Group Chimanuka were not supported based on CERVUS parentage analyses (either using strict or relaxed criteria of 95% and 80% confidence, respectively, relying on population-level allele frequencies, see Supplemental Methods S3 for more information). However, genotyping profiles of two individuals showed no mismatches to the extra-group silverback across 10–12 tested loci and were thus consistent with being related as parent–offspring. Both individuals, the subadult male Meteo and the blackback male Uhuru, interacted with the corpse. However, parentage analyses suggest that the group’s dominant silverback Chimanuka is the most likely father of Meteo based on the population-level dataset (highest LOD score) and cannot be excluded as father of Uhuru, which is also consistent with these individuals residing in the same social group. Putative mothers of both Meteo and Uhuru were former members of Group Chimanuka. Relatedness estimates derived from ML-Relate for the extra-group silverback and members of Group Chimanuka ranged from 0 to 0.5, with the highest value observed for Meteo. The two blackbacks Uhuru and Nshobole showed relatedness values of 0.2, which could be consistent with a half-sibling relationship to the extra-group silverback. No correlation was observed between the estimated relatedness values and the number of interactions with the corpse (*r* = 0.16, *df* = 19, *p* = 0.46). We thus conclude that the deceased extra-group silverback was not a first-order relative to any member of the Group Chimanuka, but relatedness at lower level cannot be excluded. Nevertheless, relatedness did not explain the frequency of interaction with the corpse.

### Overview of behavioral responses near the corpses of intra-group and extra-group individuals

**Figure 2:**
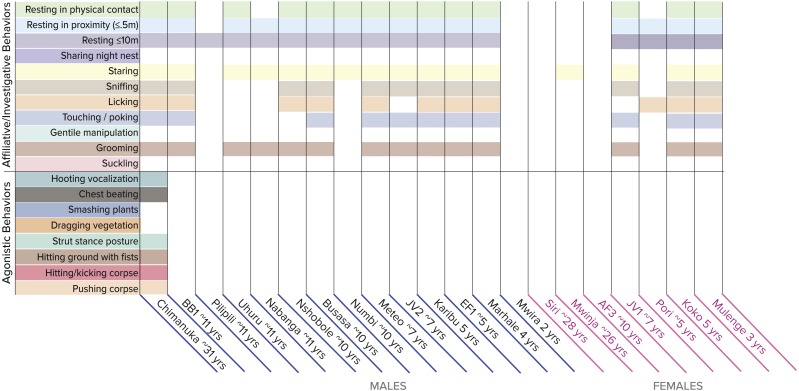
Distribution of social behaviors among individuals of different age/sex classes at an extra-group silverback’s corpse. Definitions of the behaviors are available in supplemental Table S1.

Small sample size precluded statistical analysis of the recorded behaviors, but qualitative analyses show that animals of all age groups, both sexes, and both subspecies interacted with the corpses of intra-group and extra-group individuals in some type of affiliative/investigative way, with nearly all individuals resting in contact to 10m from the corpse and staring at it (Figs. 1A–1B, Fig. 2). In the case with the corpse of the Grauer’s gorilla extra-group silverback, all individuals except the adult females, the infant male, and two blackbacks were observed sniffing, licking, touching/poking, and/or grooming the corpse (Fig. 2). The adult females were not observed at the corpse with the exception of one female that briefly stared at it while carrying her two-year-old infant on her back. In contrast to the affiliative/investigative behaviors, only silverbacks and blackbacks behaved agonistically toward the corpses of intra-group and extra-group individuals (Figs. 1A–1B and Fig. 2).

### Social dynamics in group titus before the deaths

**Figure 3:**
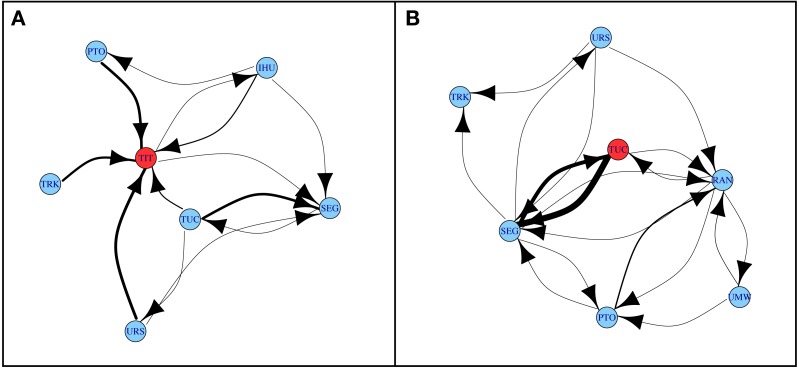
Grooming networks in Group Titus before two death events. Edge widths are proportional to the mean numbers of grooming bouts per hour and per dyad in the 12-month periods before the death of mountain gorillas Titus (A) and Tuck (B). Vertices correspond to individuals Ihumure (IHU), Pato (PTO), Segasira (SEG), Titus (TIT), Tuck (TUC), Turakora (TRK), Umushikirano (RAN), Umwana (UMW) and Urwibutso (URS).

Before Titus’ death, no dominance interactions were observed between him and Umushikirano, but Titus was an established silverback and led his group for 20 years before his death. Umushikirano had just transferred to Group Titus a month before the death. Grooming dyads during the 12 months before Titus’ death indicate that he was groomed by nearly every member of the group and he was groomed more frequently than the others (although Tuck groomed her juvenile son Segasira more often than she groomed Titus) (Fig. 3A). There were not enough data to analyze Umushikirano’s grooming relationships.

During the 12 months before Tuck’s death, no dominance interactions were observed between her and the adult female, Umwana. However, Tuck was involved in many more dominance interactions with group members compared to Umwana, suggesting Tuck occupied a more central position in the group and was the dominant female. Both adult females were subordinate to Umushikirano. Grooming relationships show that Tuck and her son Segasira groomed more often than other dyads in the group (Fig. 3B).

## Discussion

Field observations of how animals respond behaviorally to the death and corpses of conspecifics can provide valuable insights into possible ways animals gather information about the state of the individual and hence learn about death. Furthermore, a greater understanding of the extent to which animals engage with corpses may contribute substantially to our understanding of disease transmission and the amplification of outbreaks. Our observations of gorilla behavioral responses to the corpses of intra-group and extra-group members ranged from affiliative and investigative (e.g., resting in contact/proximity, licking, sniffing, poking, grooming) to agonistic (e.g., chest beating, smashing plants, hitting/kicking the corpse).

### Behavioral responses around the corpses of intra-group and extra-group individuals

Given the different social dynamics between gorillas in the same group versus between groups or with lone silverbacks, we predicted that more individuals would engage with the corpses of intra-group members than with the corpse of the extra-group silverback. Our observations were surprising because not only did almost all individuals in Grauer’s gorilla Group Chimanuka engage with the corpse of the extra-group silverback, their behavioral responses were strikingly similar to those of the mountain gorillas around the corpses of established group members. Most notably, individuals in all three cases typically sat close to the corpse and stared at it. They also touched, licked, sniffed, and groomed the corpse. In the case with the extra-group silverback, juveniles were observed to poke and lick the corpse and their fingers. The affiliative/investigative behaviors may partially be explained by a general curiosity with death. Elephants are known to be attracted to dying and dead conspecifics, regardless of genetic relatedness, group affiliations, or social relationships (Douglas-Hamilton et al., 2006; McComb, Baker & Moss, 2006). In the cases with mountain gorillas Tuck and Titus, however, their groupmates’ affiliative behaviors at their corpses may also be explained by their social relationships with group members (see more in Social Relationships section below).

In all three cases reported here, we observed only silverbacks and blackbacks behaving agonistically toward the corpses. Aggression toward corpses has been suggested to reflect an attempt to rouse the dead individual as the initial sensory information from the corpse may not always be sufficient for animals to register a permanent/irreversible change (Yao et al., 2009; Cronin et al., 2011; Wisman & Shrira, 2015). Aggression toward corpses may also be a sign of frustration at failed attempts to rouse the dead individual (Anderson, Gillies & Lock, 2010; Stewart, Piel & O’Malley, 2012). Additionally and/or alternatively, agonistic displays are typically used as a demonstration of physical strength and competitive ability by silverbacks and occasionally blackbacks during inter- and intra-group conflicts (Harcourt & Stewart, 2007). In the case of the extra-group Grauer’s gorilla silverback, the corpse may have still been perceived as a potential threat to silverback Chimanuka, especially since there were three adult females and a two-year-old infant in his group.

Since infanticide is a common reproductive strategy in gorillas, we predicted that in the case with the extra-group silverback, adult females with infants would avoid the corpse. Only one out of three adult females was observed at the corpse. She was also the only female with a dependent offspring (although she remained close to the group silverback during her brief visit). Another adult female that was not observed at the corpse was later discovered to be pregnant (she gave birth one month after the encounter). We did not observe all of the group members when we first contacted Group Chimanuka, and thus it is possible the other females visited the corpse prior to our arrival.

### Social relationships

The strength of social relationships between the surviving and deceased may explain some of the variation in individual responses toward dead conspecifics, with stronger bonds resulting in greater attendance (Stewart, Piel & O’Malley, 2012; Bercovitch, 2012). We therefore predicted that individuals who shared close social relationships with the deceased would spend more time with their corpse than would other individuals in the group. With mountain gorilla Tuck, her juvenile son, Segasira, who shared a close social relationship with her as seen through their grooming relationships and familial bond, was the individual who engaged most with her corpse. With mountain gorilla Titus, it was Ihumure, an individual known to have established a close relationship with him (and confirmed not to be his son through DNA analysis (Vigilant et al., 2015) after his mother transferred groups six months prior, who maintained closest proximity to the corpse. However, Ihumure was also severely injured during the intergroup interactions from the preceding weeks and may have been reluctant to leave Titus given his own deteriorating health. He eventually died two days after Titus. The events surrounding Titus’ death were stressful with Umushikirano’s immigration and the occurrence of several encounters with other social units. Despite the social disruption, all of the group members, including Umushikirano, still visited and rested near his corpse.

Even in the case of the extra-group silverback encountered by Grauer’s gorillas, a particular blackback, BB1, maintained a constant presence at the corpse, groomed it more than the others, and was the last animal to leave it. The extra-group silverback had not been a part of Chimanuka’s group since at least early 2014 when field staff began identifying the individuals and DNA analysis suggests he was not a close relative. We cannot exclude, however, that the two animals once knew each other.

One of the more controversial topics surrounding animal death is whether animals grieve the loss of a family member or a closely bonded group member. Among primates, especially great apes, there is compelling evidence from behavioral and physiological responses to death that they do grieve (Anderson, Gillies & Lock, 2010). Chimpanzees are known to share neuro-endocrinological circuits with humans that are activated during emotional states, such as grief (Anderson, 2011). In the case of mountain gorilla Tuck’s death, her juvenile son Segasira attempted to suckle from her corpse, despite having been weaned. This was presumably a demonstration of “comfort nursing” (Cameron, 1998), which can stimulate the release of oxytocin, a hormone that has stress inhibiting effects (Moberg & Prime, 2013). This observation, and possibly the juvenile gorilla Ihumure’s persistent proximity to mountain gorilla silverback Titus’ corpse, may suggest that humans are not unique in their capacity to grieve.

### Implications for disease transmission within and between gorilla social groups

Close inspection and direct contact with corpses present a serious risk for disease transmission. African great apes are known to be highly susceptible to the Ebola virus, with death rates among gorillas as high as 95% following contact with either the reservoir or contaminated individuals/corpses (Caillaud et al., 2006). The extent to which Ebola is transmitted directly between apes rather than primarily as spillover from a reservoir host has long been debated. Early arguments claimed group-to-group transmission was not likely a major contributor to amplifying outbreaks because direct physical contact between groups is rare (Leroy et al., 2004; Roquet et al., 2005). However, recent studies argue that the possibility of group-to-group transmission has been underestimated and direct physical contact with an infected individual/corpse is not necessarily the only means of transmission (Walsh et al., 2007). For example, final stage Ebola victims often secrete large quantities of infected bodily fluids, which could contaminate the surrounding ground and vegetation through infected saliva on discarded food remains and/or infected urine and feces left at the site. The possibility of this route of transmission could have severe implications for gorilla groups that have high home range overlap (Western lowland gorillas; Walsh et al., 2007; Grauer’s gorillas; Yamagiwa et al., 2012; mountain gorillas; Caillaud et al., 2014). Furthermore, in Western lowland gorillas, different social groups frequently feed in the same fruiting trees in close temporal succession (Walsh et al., 2007). Tissues from wild ape carcasses have also been shown to yield viable Ebola virus for up to three days after death (Leroy et al., 2004). The stability of the virus suggests that there is ample time for other groups to discover infectious corpses.

The close inspection of corpses by almost every group member in all three of our cases suggest that direct ape-to-ape transmission as well as group-to-group transmission could play a critical role in disease outbreak amplification among gorillas. In the case with the extra-group silverback, several animals inspected the vegetation surrounding the corpse with their hands and mouth and sniffed the secreted feces of the deceased. The feces contained a large quantity of *Myrianthus* seeds, a preferred fruit by both the Grauer’s gorillas and the sympatric eastern chimpanzees (*Pan troglodytes schweinfurthii*), which are known to overlap in their diet and ranging with the gorillas in Kahuzi-Biega (Yamagiwa et al., 2012), adding to the possibility of cross species contamination.

## Conclusion

Given the severe health risks that can accompany close inspection of corpses, it is curious why the behavior occurs so widely among animals, especially primates. Close inspection of corpses could be an example of antagonistic pleiotropy, which can result in both beneficial and detrimental effects (e.g., increased fitness through the acquisition of valuable information, such as danger in the environment or the loss of a competitor, which could then lead to the acquisition of new mates but with the trade-off of increased susceptibility to infectious diseases). It is also possible that opportunities to interact with corpses are rare (with the exception of infants), resulting in a weak selection pressure against it.

It remains unclear whether the behaviors we observed around the corpses in these three cases are a common response in gorillas. Since 2004, we have documented 42 mountain gorilla deaths from individuals older than 3.5 yrs. In 22 of the 42 cases, the ailing individual either left the group to die alone or was abandoned when it could no longer keep up with the group. In 17 other cases, the dead gorilla was last seen alive with the group, and thus it could not be excluded that group members interacted with the corpse before it was detected. Future studies should pay special attention to the frequency with which corpses are encountered and to the circumstances that lead to group members being abandoned before death and those that receive prolonged attendance.

##  Supplemental Information

10.7717/peerj.6655/supp-1 Table S1 Definitions of behaviors recorded around gorilla corpses

10.7717/peerj.6655/supp-2 Table S2 Estimates of genetic diversity per locus within the Kahuzi-Biega National Park population of Grauer’s gorillas A, Observed number of alleles, H _*O*_, observed heterozygosity, H _*E*_, expected heterozygosity.

10.7717/peerj.6655/supp-3 Data S1 Genotyping and parentage assignment using Cervus v.3.0.7 Description of the method used to assign parentage to Grauer’s gorillas from Group Chimanuka.

## Additional Information and Declarations

### Field Study Permissions

The following information was supplied relating to field study approvals (i.e., approving body and any reference numbers): The study was conducted in compliance with legal requirements of the Republic of Rwanda and Democratic Republic of Congo. The data collection protocols were approved by the Rwandan Development Board and by the Institut Congolais pour la Conservation de la Nature.

### Data Availability

The following information was supplied regarding data availability: Our videos and photographs are available through Figshare: Porter, Amy; Eckardt, Winnie; Vecellio, Veronica; Guschanski, Katerina; Niehoff, Peter; Ngobobo, Urbain; et al. (2018): Mountain gorilla juvenile male Segasira with his mother Tuck’s corpse.mp4. figshare. Media. https://doi.org/10.6084/m9.figshare.6198584.v1
Porter, Amy; Eckardt, Winnie; Vecellio, Veronica; Guschanski, Katerina; Niehoff, Peter; Ngobobo, Urbain; et al. (2018): Mountain gorilla juvenile male Segasira with his mother Tuck’s corpse. figshare. Figure. https://doi.org/10.6084/m9.figshare.6198587.v1
Porter, Amy; Eckardt, Winnie; Vecellio, Veronica; Guschanski, Katerina; Niehoff, Peter; Ngobobo, Urbain; et al. (2018): Mountain gorilla blackback Urwibutso displays with his mother Tuck’s corpse. figshare. Media. https://doi.org/10.6084/m9.figshare.6198590.v1
Porter, Amy; Eckardt, Winnie; Vecellio, Veronica; Guschanski, Katerina; Niehoff, Peter; Ngobobo, Urbain; et al. (2018): Mountain gorilla silverback Umushikirano displays with adult female Tuck’s corpse. figshare. Media. https://doi.org/10.6084/m9.figshare.6198593.v1
Porter, Amy; Eckardt, Winnie; Vecellio, Veronica; Guschanski, Katerina; Niehoff, Peter; Ngobobo, Urbain; et al. (2018): Grauer’s gorilla silverback Chimanuka displays with the corpse of an extra-group silverback. figshare. Media. https://doi.org/10.6084/m9.figshare.6198596.v1
Porter, Amy; Eckhardt, Winnie; Vecellio, Veronica; Guschanski, Katerina; Niehoff, Peter; Ngobobo, Urbain; et al. (2018): Grauer’s gorilla affiliative and agonistic behaviors toward the corpse of an extra-group silverback. figshare. Media. https://doi.org/10.6084/m9.figshare.6198599.v1
Porter, Amy; Eckardt, Winnie; Vecellio, Veronica; Guschanski, Katerina; Niehoff, Peter; Ngobobo, Urbain; et al. (2018): Grauer’s gorilla group Chimanuka gathered around the corpse of an extra-group silverback. figshare. Figure. https://doi.org/10.6084/m9.figshare.6198602.v1
Porter, Amy; Eckhardt, Winnie; Vecellio, Veronica; Guschanski, Katerina; Niehoff, Peter; Ngobobo, Urbain; et al. (2018): Grauer’s gorilla blackback named BB1 grooming the corpse of an extra-group silverback. figshare. Figure. https://doi.org/10.6084/m9.figshare.6198605.v1
Raw data are available in MS Excel format through Figshare: Porter, Amy; Eckardt, Winnie; Vecellio, Veronica; Guschanski, Katerina; Niehoff, Peter; Ngobobo, Urbain; et al. (2019): Behavioral responses of mountain gorillas around the corpse of a dominant group silverback, Titus. figshare. Dataset. https://doi.org/10.6084/m9.figshare.7616633
Porter, Amy; Eckhardt, Winnie; Vecellio, Veronica; Guschanski, Katerina; Niehoff, Peter; Ngobobo, Urbain; et al. (2019): Behavioral responses of mountain gorillas around the corpse of a dominant group adult female, Tuck. figshare. Dataset. https://doi.org/10.6084/m9.figshare.7616714
Porter, Amy; Eckhardt, Winnie; Vecellio, Veronica; Guschanski, Katerina; Niehoff, Peter; Ngobobo, Urbain; et al. (2019): Behavioral responses of Grauer’s gorillas around the corpse of an extra-group silverback. figshare. Dataset. https://doi.org/10.6084/m9.figshare.7616837
Porter, Amy; Eckhardt, Winnie; Vecellio, Veronica; Guschanski, Katerina; Niehoff, Peter; Ngobobo, Urbain; et al. (2019): Titus total observation times. figshare. Dataset. https://doi.org/10.6084/m9.figshare.7622312
Porter, Amy; Eckardt, Winnie; Vecellio, Veronica; Guschanski, Katerina; Niehoff, Peter; Ngobobo, Urbain; et al. (2019): Tuck total observation times. figshare. Dataset. https://doi.org/10.6084/m9.figshare.7622378
Porter, Amy; Eckardt, Winnie; Vecellio, Veronica; Guschanski, Katerina; Niehoff, Peter; Ngobobo, Urbain; et al. (2019): Titus grooming bout counts. figshare. Dataset. https://doi.org/10.6084/m9.figshare.7622408.v1
Porter, Amy; Eckardt, Winnie; Vecellio, Veronica; Guschanski, Katerina; Niehoff, Peter; Ngobobo, Urbain; et al. (2019): Tuck grooming bout counts. figshare. Dataset. https://doi.org/10.6084/m9.figshare.7622504.v1
Porter, Amy; Eckhardt, Winnie; Vecellio, Veronica; Guschanski, Katerina; Niehoff, Peter; Ngobobo, Urbain; et al. (2019): Titus displace and avoidance counts. figshare. Dataset. https://doi.org/10.6084/m9.figshare.7622573.v1
Porter, Amy; Eckardt, Winnie; Vecellio, Veronica; Guschanski, Katerina; Niehoff, Peter; Ngobobo, Urbain; et al. (2019): Tuck displacement and avoidance counts. figshare. Dataset. https://doi.org/10.6084/m9.figshare.7622642.v1.
