# Supplementary material for: Behavioral responses around conspecific corpses in adult eastern gorillas (Gorilla beringei spp.)
Source: PeerJ. 2019 Apr 2;7:e6655. doi: 10.7717/peerj.6655 (PMC6450378; doi:10.7717/peerj.6655)
Supplement: Table S1 [file peerj-07-6655-s001.pdf]

Table S1: Definitions of behaviors recorded around gorilla corpses

Type	Behavior	Definition
Affiliative	Resting in physical contact	A gorilla remains stationary while sitting or lying in contact with the corpse
	Resting in proximity (≤0.5m)	A gorilla remains stationary while sitting or lying ≤0.5m from the corpse
	Resting ≤10m	A gorilla remains stationary while sitting or lying ≤10m from the corpse.
	Sharing night nest	Two or more gorillas use the same nest to sleep in through the night
	Gentle Manipulation	A gorilla gently moves a body part on the corpse
	Grooming	A gorilla picks through the fur of the corpse with fingers or lips to remove items (e.g., dry skin, dirt, insects)
	Suckling	A gorilla has its mouth in contact with a nipple of a female while sucking on it
Affiliative / Investigative	Staring	A gorilla looks intently at the corpse
	Sniffing	A gorilla smells the corpse from close (0-0.5m) range
	Licking	A gorilla touches the corpse with his/her tongue
	Touching/Poking	A gorilla places a hand/foot/finger on the corpse. Poking is sometimes followed by licking the finger that touched the corpse
Agonistic	Hooting vocalization	Definition from Fossey (1972) Usually a series of vocalizations "given with or without a terminating chest-beat, consists of prolonged distinct *hoo-hoo-hoos*. Low pitched, often undetectable to human ear at beginning, but usually built up to plaintive-sounding and longer hoots toward the end"
	Chest beating	Striking the chest rapidly and repeatedly, alternating with both hands
	Smashing plants	Often used in displays toward other gorillas, an animal uses hands/feet to forcefully push down vegetation
	Dragging vegetation	Often used in displays toward other gorillas, an animal grabs vegetation with the hand and drags it while moving
	Strut stance posture	Quadrupedal posture with back arched, head up, lips compressed tightly, limbs straight and stiff
	Hitting ground w/ fists	Pounding the ground forcefully with one or two fists while standing or moving
	Hitting/kicking corpse	A gorilla uses hands/feet to hit/kick the corpse
	Pushing corpse	A gorilla uses hands to forcefully move the corpse

Reference:

Fossey D. 1972. Vocalizations of the mountain gorilla (*Gorilla gorilla beringei*). *Animal Behaviour* 20:36-53.
