# Supplementary material for: Behavioral responses around conspecific corpses in adult eastern gorillas (Gorilla beringei spp.)
Source: PeerJ. 2019 Apr 2;7:e6655. doi: 10.7717/peerj.6655 (PMC6450378; doi:10.7717/peerj.6655)
Supplement: Table S2 — A, Observed number of alleles, H O, observed heterozygosity, H E, expected heterozygosity. [file peerj-07-6655-s002.pdf]

Table S2: Estimates of genetic diversity per locus within the Kahuzi-Biega National Park population of Grauer's gorillas.

**Locus**	**A**	**H_O**	**H_E**
vWF	5	0.517	0.469
D1s550	4	0.75	0.69
D4s1627	5	0.609	0.559
D5s1457	6	0.708	0.734
D5s1470	5	0.708	0.745
D6s474	6	0.578	0.57
D6s1056	6	0.689	0.691
D7s817	5	0.606	0.571
D8s1106	5	0.578	0.598
D10s1432	6	0.684	0.697
D14s306	4	0.659	0.695
D16s2624	3	0.505	0.469

A: Observed number of alleles, H_O: observed heterozygosity, H_E: expected heterozygosity
