# Supplementary material for: Behavioral responses around conspecific corpses in adult eastern gorillas (Gorilla beringei spp.)
Source: PeerJ. 2019 Apr 2;7:e6655. doi: 10.7717/peerj.6655 (PMC6450378; doi:10.7717/peerj.6655)
Supplement: Data S1 — Description of the method used to assign parentage to Grauer’s gorillas from Group Chimanuka. [file peerj-07-6655-s003.pdf]

Supplemental Method S3: Genotyping and parentage assignment

The genotypes of 149 samples corresponded to 96 unique individuals based on the identity analysis performed in CERVUS v 3.0.7 (Kalinowski, Taper & Marshall, 2007). After merging the genotypes that belong to the same individual into a consensus genotype based on identical genotypes at minimum of five loci, the individual genotypes were 97% complete. The ability to determine parent-offspring relationships within this dataset was high, with a combined non-exclusion probability of 0.048 for only one parent.

The deceased extra-group silverback and the members of the Group Chimanuka were analyzed for the presence of genotypic incompatibilities (*i.e.*, mismatches) to check for the likelihood of parent-offspring relationships using CERVUS. All pairwise comparisons with females showed at least one mismatch, whereas no mismatches were found between the extra-group silverback and two males within Group Chimanuka, the subadult Meteo and the young adult male Uhuru.
